# *YAP1* amplification as a prognostic factor of definitive chemoradiotherapy in nonsurgical esophageal squamous cell carcinoma

**DOI:** 10.1002/cam4.2761

**Published:** 2019-12-18

**Authors:** Honghai Dai, Yang W. Shao, Xiaoling Tong, Xue Wu, Jiaohui Pang, Alei Feng, Zhe Yang

**Affiliations:** ^1^ Tumor Research and Therapy Center Shandong Provincial Hospital Affiliated to Shandong University Jinan China; ^2^ Nanjing Geneseeq Technology Inc Nanjing China; ^3^ School of Public Health Nanjing Medical University Nanjing China; ^4^ Translational Medicine Research Institute Geneseeq Technology Inc Toronto Ontario Canada

**Keywords:** chemoradiation therapy, esophageal squamous cell carcinoma, local recurrence‐free survival, next‐generation sequencing, *YAP1* amplification

## Abstract

**Background:**

Definitive chemoradiation therapy (dCRT) is the standard treatment for patients with nonsurgical esophageal squamous cell carcinoma (ESCC), yet patients have demonstrated great variations in their responses to dCRT and inevitably progressed following treatment.

**Methods:**

To identify prognostic biomarkers, we performed targeted next‐generation sequencing of 416 cancer‐related genes on primary tumors from 47 nonsurgical ESCC patients prior to dCRT treatment. The association between genetic alterations and patients' local recurrence‐free survival (LRFS), progression‐free survival (PFS), and overall survival (OS) was analyzed.

**Results:**

*TP53* (78% of patients), *NOTCH1* (32%), *ARID1A* (13%), *FAT1* (13%), and *CDKN2A* (13%) were commonly mutated in ESCC patients, while gene amplifications frequently occurred in *MCL1* (36%), *FGF19* (34%), *MYC* (32%), *CCND1* (27%), *ZNF217* (15%), *CDKN2A* (13%), and *YAP1* (11%). Univariate and multivariate analyses of clinical factors and genetic alterations indicated that sex is an independent prognostic factor, with males tending to have better LRFS (hazard ratio [HR], 0.25; 95%CI, 0.08‐0.77, *P* = .015) and progression‐free survival (PFS) (HR, 0.35; 95%CI, 0.13‐0.93, *P* = .030) following dCRT. Meanwhile, *YAP1* amplification (n = 7) was an adverse prognostic factor, and patients with this alteration demonstrated a tendency toward worse outcomes with shorter LRFS (HR, 4.06; 95%CI, 1.26‐13.14, *P* = .019) and OS (HR, 2.78; 95%CI, 0.95‐8.17, *P* = .062). In a subgroup analysis, while sex and M‐stage were controlled, a much stronger negative effect of *YAP1* amplification vs wild‐type in LRFS was observed (log‐rank *P* = .0067).

**Conclusion:**

The results suggested that *YAP1* amplification is a potentially useful biomarker for predicting treatment outcomes and identifying patients with a high risk of relapse who should be closely monitored.

## Graphical abstract

We found that in patients with nonsurgical esophageal squamous cell carcinoma, YAP1 amplification is an adverse prognostic factor associated with significantly shorter local recurrent‐free survival (LRFS) after definitive chemoradiotherapy.

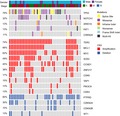

## 1 INTRODUCTION

Esophageal cancer is one of the most common malignancies worldwide, accounting for 4% of cancer‐related deaths in the US and 13% in China.^1^, ^2^, ^3^ Its two major histological subtypes, adenocarcinoma and squamous cell carcinoma (SCC), are distributed differently between different ethnic groups. Adenocarcinoma is the dominant subtype in Europe and North America, while ESCC is more prevalent in Asia, Africa, and South America.^4^ Approximately 40%‐60% of patients with ESCC are inoperable at the time of diagnosis due to locally advanced disease, distant metastasis, poor performance status, and the existence of comorbidities that increase surgical risks.^5^ For those patients, definitive chemoradiation therapy (dCRT) is the standard curative therapy and has demonstrated efficacy in improving overall survival (OS) time and locoregional control rate over radiotherapy alone, or the sequential use of radiotherapy and chemotherapy.^6^, ^7^, ^8^, ^9^ Although dCRT results in relatively high response rates and favorable short‐term survival, the locoregional recurrence rate of ESCC is high (40%‐55%) within 5 years of treatment. Distant recurrence also occurred in 28% ESCC patients.^10^, ^11^ For patients who developed local recurrence, 50% experienced the first recurrence within 6 months after dCRT, which was highly correlated with a poorer prognosis.^11^, ^12^

Previous studies have identified several prognostic factors for poor treatment outcomes of dCRT, such as lymph node metastasis,^13^ pretreatment weight loss,^14^ history of heavy smoking,^15^ and an increased number of circulating tumor cells prior to chemoradiotherapy.^16^ However, none of the previous studies explored the genetic biomarkers that could possibly correlate with the prognosis of dCRT. To identify potential biomarkers for discriminating patients at a heightened risk of early failure and poor prognosis, we conducted this retrospective study by performing targeted next‐generation sequencing (NGS) of 416 cancer‐related genes on pre‐dCRT tumor biopsies in 47 unresectable ESCCs, and correlating those genetic alterations to the treatment outcomes following dCRT.

## 2 MATERIALS AND METHODS

### 2.1 Patients

The study included 47 patients who were diagnosed with ESCC and underwent dCRT in the Oncology Center of Shandong Provincial Hospital. The inclusion criteria were as follows: (a) all patients had histologically proven primary ESCC; (b) the diseases were classified as stage II‐III and IV with only supraclavicular lymph node metastasis, according to the 7th edition of the American Joint Committee on Cancer [AJCC] staging system for ESCC^17^; (c) all patients were precluded from surgery and received dCRT; (d) the Eastern Cooperative Oncology Group (ECOG) performance score ranged from 0 to 2; and (e) patients had adequate bone marrow, renal, and hepatic functions to endure the treatment. The study was approved by the Ethical Review Board of the Oncology Center of Shandong Provincial Hospital, and informed written consent was obtained from all participants or their next of kin, if the patient was diseased.

### 2.2 dCRT treatment approaches

All patients received standard dCRT. A median of two cycles of fluorouracil and cisplatin were administrated concurrently with radiotherapy. For radiotherapy, three‐dimensional conformal radiotherapy (3DCRT) and intensity‐modulated radiotherapy (IMRT) were used, and the target volume was based on the clinical target volume (CTV), which included an expansion of 3‐4 cm above and below the gross tumor and corresponding lymphatic drainage area. The planning target volume (PTV) was defined as the CTV plus a 0.8 cm expansion margin. All patients received a total radiation dose of 60‐66 Gy with 2 Gy per fraction, 5 days per week.

### 2.3 Evaluations and outcomes

The follow‐up of all patients was conducted 1 month after radiotherapy, and every 3 months thereafter during the first year. After the first year, patients were followed up with every 3‐6 months. Disease responses were evaluated according to the revised RECIST (Response Evaluation Criteria in Solid Tumors) guideline, version 1.1.

Treatment responses were monitored by esophagography and CT imaging at each follow‐up evaluation, and compared to the images obtained prior to dCRT or those from the preceding follow‐up. Suspected esophageal recurrences were confirmed by histological or cytological testing of tumor biopsies. Lymph node recurrences were diagnosed when lymph nodes reappeared after complete disappearance, or became enlarged after remaining stable, or newly enlarged lymph nodes were detected. Suspected supraclavicular lymph node recurrences were confirmed by fine‐needle aspiration and pathological examination. Disease recurrence was classified as locoregional and/or distant. Locoregional recurrence included tumor recurrences at the site of the primary tumor or locoregional lymph nodes. Distant recurrence included nonregional lymph node recurrences, systemic metastases, malignant pleural effusions, and peritoneal metastases.^18^

OS in this study was defined from the beginning of radiotherapy to the time of death from any cause, or the date of last follow‐up evaluation. PFS was defined from the beginning of radiotherapy to the time of tumor recurrence, or death in the absence of tumor recurrence, or the date of last follow‐up evaluation. LRFS was defined from the beginning of radiotherapy to the time of tumor recurrences at the site of the primary tumor or locoregional lymph nodes recurrence, or death in the absence of primary tumor or locoregional lymph nodes recurrence, or the date of the last follow‐up evaluation.

### 2.4 DNA extraction and sequencing library preparation

For each patient, archived formalin‐fixed and paraffin‐embedded (FFPE) blocks of tumor biopsies that were collected through endoscopic inspection before the dCRT were used for DNA extraction. The tumor content of all samples was confirmed to be at least 10% by pathologists. Eight 10‐µm FFPE sections were de‐paraffinized with xylene, from which genomic DNA was extracted using the QIAamp DNA FFPE Tissue Kit (Qiagen) according to the manufacturer's protocol. The quantity and quality of the extracted DNA were evaluated using a Qubit 3.0 fluorometer and Nanodrop 2000, respectively (Thermo Fisher Scientific). The DNA was fragmented using a Covaris M220 sonication system to obtain 350 bp fragments and purified using Agencourt AMPure XP beads (Beckman Coulter).

Library preparations of the fragmented DNA were performed using the KAPA hyper library preparation kit (KAPA Biosystems), following the manufacturer's protocol. Libraries with different indices were pooled for targeted enrichment with IDT xGen Lockdown Reagents, and a customized enrichment panel (IDT) covering the exonic regions of 416 genes and the introns of 16 fusion genes. The captured library was further amplified using Illumina p5 (5' AAT GAT ACG GCG ACC ACC GA 3') and p7 (5' CAA GCA GAA GAC GGC ATA CGA GAT 3') primers in the KAPA Hifi HotStart ReadyMix (KAPA Biosystems), and purified with Agencourt AMPure XP beads. Sequencing libraries were quantified by qPCR using the KAPA Library Quantification kit (KAPA Biosystems) and the size distribution was examined on a Bioanalyzer 2100 (Agilent Technologies). The final libraries were sequenced on an Illumina Hiseq 4000 platform to a mean coverage depth of at least 250×, following the manufacturer's instructions.

### 2.5 Sequencing data analysis

The sequencing data were analyzed by a validated automation pipeline with the main steps being performed as previously described.^19^ Data cleaning was performed in bck2fastq for demultiplexing and then Trimmomatic^20^ was used for FASTQ file quality control (QC). Leading/trailing low quality (base phred score below 30) or N bases were removed. Read mapping to the reference human genome, hg19, was conducted in the Burrows‐Wheeler Aligner (BWA‐mem, v0.7.12).^21^ PCR duplicates were removed by Picard. The Genome Analysis Toolkit (GATK 3.4.0) was employed to apply local realignment around indels and recalibrate the base quality score. VarScan2 was employed for the detection of single‐nucleotide variations (SNVs) and insertion/deletion mutations with the following parameters: minimum read depth = 20, minimum base quality = 25, minimum variant allele frequency (VAF) = 0.03, minimum variant supporting reads = 3, variant supporting reads mapped to both strands, and strand bias no greater than 10%.^22^

A comprehensive assay validation was performed for the copy number variation (CNV) pipeline using 38 samples against droplet digital polymerase chain reaction (ddPCR) results as the “gold standard.” We reduced system noise in copy number data by principal component analysis of 100 normal samples sequenced in the same batch. This analysis pipeline is capable of detecting 0.4‐fold copy number loss at ≥50% tumor cell content, and fourfold amplification at ≥10% tumor cell content. A ≥ 1.6‐fold change in DNA copy number was set as the cutoff for amplification, while a ≤ 0.6‐fold change was used as the cutoff for deletion.

### 2.6 Variant filtering and annotation

The vcf files containing both single‐nucleotide polymorphism (SNP) and small insertions/deletions (indels) were annotated by ANNOVAR against the following databases: dbSNP (v138), 1000 Genome, ExAC, COSMIC (v70), ClinVAR, and SIFT. Mutations were removed if they were present in a > 1% population frequency in the 1000 Genomes Project or 65000 exomes project (ExAC). The resulting mutation lists were filtered through an internally collected list of recurrent sequencing errors on the same sequencing platform, which was compiled from the sequencing results of 53 normal samples with a minimum average sequencing depth of 700×. Specifically, if a variant was detected (ie, ≥3 mutant reads and > 1% VAF) in > 20% of the normal samples, it was considered a likely artifact and was removed. Mutations occurring within the repeat masked regions were also removed. In an additional filtering step, a mutation was only called out when the VAF was above 2% with a minimum of three mutant reads for COSMIC mutations, or above 3% with a minimum of five mutant reads for non‐COSMIC mutations.

### 2.7 Statistical analysis

The median and 95% confidence intervals were calculated using the Wilcoxon signed‐rank test. Univariate and multivariate analyses using the Cox model were performed to calculate the associations between patients' clinical characteristics—including age, sex, disease stage, tumor length, and smoking history—and LRFS, OS, and PFS. The Kaplan‐Meier method was used to calculate survival rates, and the log‐rank test was used to analyze differences between groups. A statistically significant difference was set at *P* < .05.

## 3 RESULTS

### 3.1 Patient characteristics and treatment outcomes

The clinical characteristics of the 47 patients included in this study are shown in Table 1. The median age of the patients was 64 years, and ranged from 41 to 83 years. Thirty‐eight patients (80.9%) were male and nine (19.1%) were female. The majority of patients had stage II (15, 31.9%) or III (28, 59.6%) disease, and only four patients had stage IV disease with supraclavicular lymph node metastasis. Heavy smokers (smoke ≥ 20/day or ≥ 20 packs/year) accounted for 68.1% of all patients (n = 32).

**Table 1:** Clinical characteristics and treatment outcomes of the 47 ESCC patients

Characteristics		Value or No. of Patients (%)
Age (year) Median (range) 64 (41‐83)		
41‐50		7 (14.9)
51‐60		12 (25.5)
61‐70		20 (42.6)
>70		8 (17.0)
Sex		
Male		38 (80.9)
Female		9 (19.1)
Smoking history		
Heavy smoker		32 (68.1)
Nonsmoker		15 (31.9)
TNM stage (2009 AJCC)		
T‐stage	2	33 (70.2)
	3	5 (10.6)
	4	9 (19.1)
N‐stage	0	16 (34.0)
	1‐3	31 (66.0)
M‐stage	0	43 (91.5)
	1	4 (8.5)
Tumor length (cm)		
≤3		8 (17.0)
>3		39 (83.0)
Failure categories		
Local failure		20 (42.6)
Distant failure		10 (21.3)
No evidence of relapse		17 (36.2)
Overall best response		
CR		25 (53.2)
PR		10 (21.3)
SD		8 (17.0)
PD		4 (8.5)

Abbreviations: CR, complete response; PD, progression disease; PR, partial response; SD, stable disease.

All patients were followed until death or the end of the study, which ranged from 5.9 to 106.5 months, with a median of 19.4 months (Table 1). At the time of the last follow‐up, 16 (34%) patients were alive. Of all patients, 20 (42.6%) had local recurrences, 10 (21.3%) had distant diseases, and 17 (36.2%) had no detectable tumor recurrences. The median PFS time was 9.8 months with the occurrence of either local or distance relapse (Table 1).

### 3.2 Gene amplifications are the predominant features of ESCC

**Figure 1:**
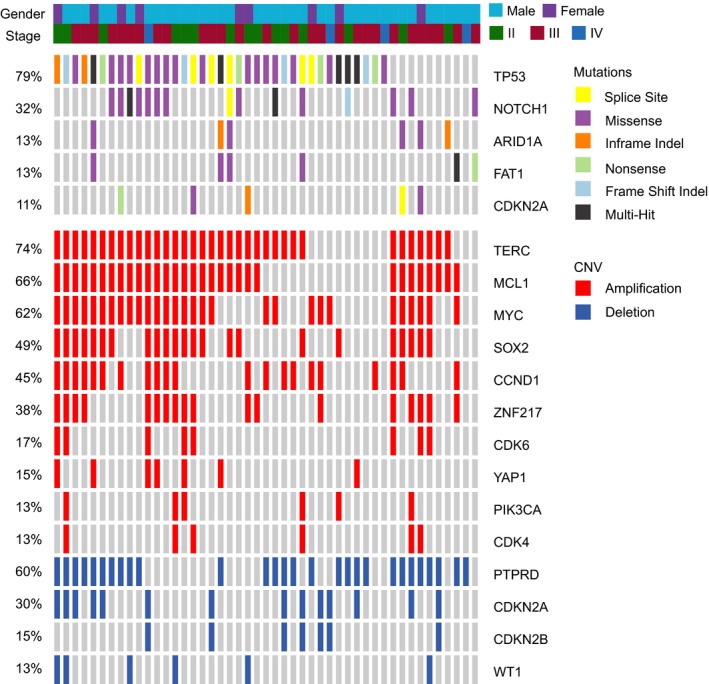
Mutation and copy number variation (CNV) plot for all patients. Each column represents one patient, and only genes that have alterations in > 5 patients are shown

We performed targeted NGS of 416 cancer‐related genes on treatment‐naïve tumor biopsies from 47 ESCC patients. Genes that were mutated in more than five cases (>10% patients) included *TP53* (79%), *NOTCH1* (32%), *ARID2A* (13%), *FAT1* (13%), and *CDKN2A* (11%), which were consistent with a previous report.^23^ *TP53* mutations were identified in 38 patients, among which 20 patients had frameshift or nonsense mutations (Table S1). Our analysis showed that (CNVs) were prevalent in the cohort, with 46 (98%) of patients having CNVs in one or more genes (Figure 1). The most common genes with CNVs were located on chromosomes 3q26 (*TERC, SOX2, PIK3CA*), 11q13 (*CCND1, FGF19*), 11q22 (*YAP1*), 1q21 (*MCL1*), and 8q24 (*MYC, PTK2, RECQL4*). *TERC*, which encodes an RNA component of telomerase and serves as the template of the telomere, was amplified in 35 patients (74%), while *SOX2*, another gene on chromosome 3q26, was amplified in 23 patients (47%), and 23 patients exhibited amplification of both genes (Figure 1). Other frequently amplified genes included *MCL1* (66% of patients), *MYC* (62%), *CCND1* (45%), *ZNF217* (38%), CDK6 (17%), and *YAP1* (15%), which are all modulators of cell cycle,^24^ apoptosis,^25^ proliferation, or the cytoskeleton.^26^, ^27^, ^28^

### 3.3 The prognostic role of genetic alterations in disease recurrence

Clinical factors and genetic alterations are all potential predictors of prognosis in dCRT treatment. Therefore, we examined the association of the top gene alterations in Figure 1 with patients' LRFS, PFS, and OS using the univariate Cox regression model. Baseline clinical characteristics, including age, sex, TNM stages, tumor length, and smoking history, were examined together. Variables with *P* < .2 were then used for multivariate analyses. Sex was found to be an independent predictor of LRFS (HR 0.25, *P* = .015) and PFS (HR 0.35, *P* = .030), with poorer outcomes in females compared to males. Meanwhile, advanced M‐stage was associated with poorer LRFS (HR 4.35, *P* = .030, Table 2) than early stage disease. Multivariate analyses revealed that *YAP1* amplification was the only genetic biomarker found to be significantly associated with shorter LRFS (HR 4.06, *P* = .019). A nearly significant association was also found between *YAP1* amplification and shorter OS (HR 2.78, *P* = .062), while a nonsignificant association was observed with PFS (HR 1.56, *P* = .11, Table 2).

**Table 2:** Univariate and multivariate Cox regression analyses of prognostic parameters. Variables with a *P*‐value < 0.2 in univariate tests were selected for multivariate tests

		Univariate			Multivariate		
		HR	95% CI	*P‐*value	HR	95% CI	*P*‐value
LRFS	Sex (male)	0.35	0.13‐0.96	.042^b^	0.25	0.08‐0.77	.015^b^
	M‐stage	3.6	1‐13	.043^b^	4.35	1.15‐16.46	.030^b^
	*CCND1* amp	0.48	0.18‐1.3	.14	0.53	0.16‐1.70	.284
	*YAP1* amp	2.9	1‐8.3	.046^b^	4.06	1.26‐13.14	.019^b^
	*ZNF217* amp	0.45	0.16‐1.3	.13	0.45	0.14‐1.50	.195
PFS	Age	0.97	0.93‐1.00	.08	0.96	0.92‐1.00	.031^b^
	Sex (male)	0.49	0.20‐1.20	.11	0.35	0.13‐0.93	.030^b^
	*YAP1* amp	2.10	0.85‐5.30	.11	1.56	0.62‐4.02	.303
OS	Sex (male)	0.48	0.2‐1.1	.1	0.43	0.18‐1.06	.067^a^
	*FAT1* mutation	1.9	0.73‐5.1	.19	1.46	0.47‐4.47	.513
	*YAP1* amp	2.3	0.91‐5.8	.078	2.78	0.95‐8.17	.062^a^
	*ZNF217* amp	0.59	0.27‐1.3	.18	0.49	0.21‐1.16	.105

Abbreviation: amp, amplification.

a Close to statistically significant

b Statistically significant.

### 3.4 The predictive role of YAP1 amplification in ESCC prognosis

**Figure 2:**
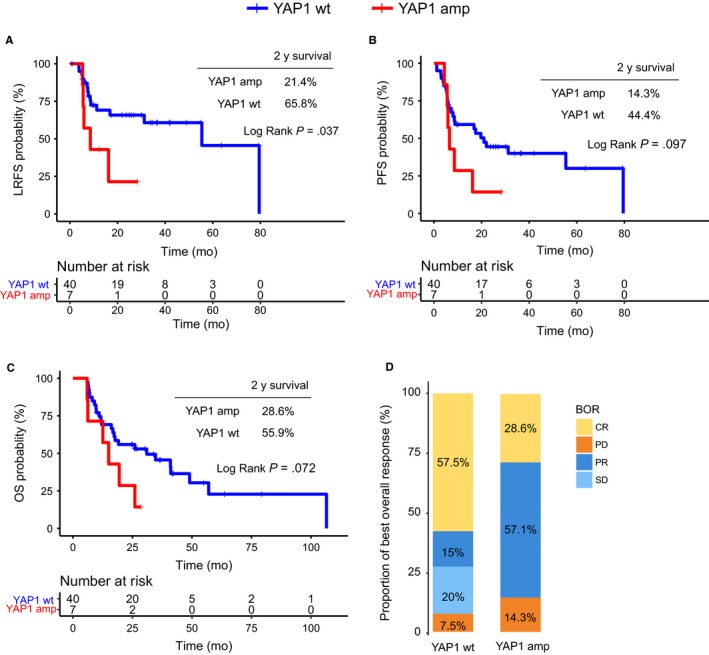
Survival analysis and best overall responses (BORs) in patients with YAP1 amplification. A‐C, Kaplan‐Meier survival curves for local recurrence‐free survival (LRFS), progression‐free survival (PFS), and overall survival (OS) for the 47 ESCC patients. D, The BOR of all patients in the *YAP1* wt and amplification groups

The prognostic effect of *YAP1* CNV on LRFS, PFS, and OS was illustrated by Kaplan‐Meier curves (Figure 2). The data showed that patients with wild‐type (wt) *YAP1* had a significantly better LRFS (median: 55.4 months), compared to patients with *YAP1* amplification (median: 8.6 months, *P* = .037, Figure 2A). The 2‐year survival probability was also different between the two groups (wt vs amplification: 65.8% vs 21.4%). Moreover, favorable PFS and OS were observed in patients with wt *YAP1*, despite that such differences were not significant (PFS *P* = .097; OS *P* = .072; Figure 2B,C). The best overall responses (BORs) were also different between the two groups, with a lower complete response (CR, 28.6% vs 57.5%) in the *YAP1* amplification group, compared to *YAP1* wt group, which might be associated with increased locoregional recurrence following dCRT (Figure 2D).

**Figure 3:**
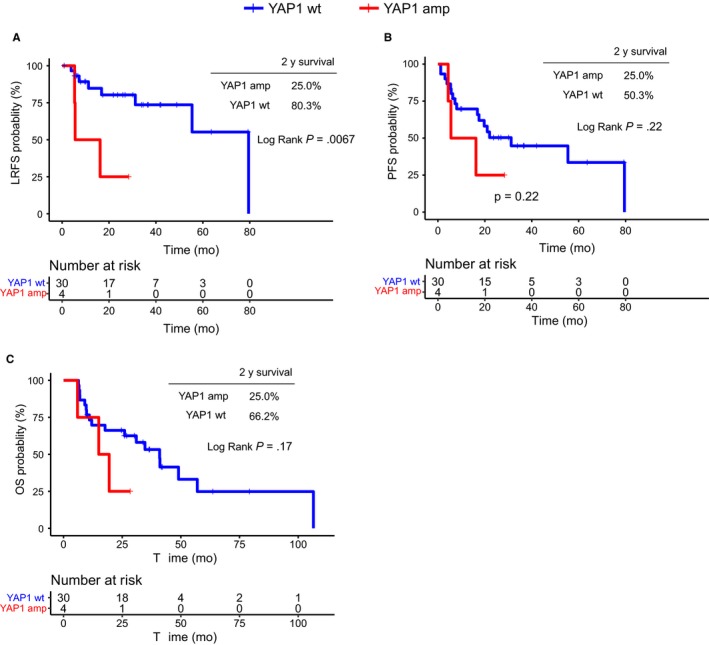
Kaplan‐Meier survival curves for local recurrence‐free survival (LRFS), progression‐free survival (PFS), and overall survival (OS) in male patients with stage II‐III disease (n = 34)

Given that both M staging and sex were associated with LRFS in multivariate analyses (Table 2), we compared the survival times of male patients with stage II‐III disease between the *YAP1* amplification and wt groups. As illustrated in Figure 3A, after controlling for clinical stage and sex, the survival advantage become even more pronounced for *YAP1* wt patients. Such patients demonstrated significant improvements in LRFS (wt vs amplification median: 79.5 vs 10.9 months, *P* = .0067, Figure 3A), and visually better PFS (31.2 vs 10.9 months, *P* = .22, Figure 3B) and OS (40.9 vs 17.2 months, *P* = .17, Figure 3C), compared to *YAP1* amplification patients. The prognostic role of *YAP1* amplification in female patients was also examined. However, due to the limited number of patients (n = 2), we only observed a trend for better OS in YAP1 wt patients vs YAP1 amplification patients, while the difference in LRFS and PFS could not be discriminated between the groups (Figure S1).

## 4 DISCUSSION

In this study, using a pan‐cancer NGS panel, we retrospectively inspected the cancer genomes of 47 patients with inoperable ESCC, with the aim of identifying prognostic genetic biomarkers for dCRT. Baseline clinical features were accounted for in the analysis and sex appeared to be an independent prognostic factor in LRFS and PFS, with increased HR in females. The lower impact of sex on OS is consistent with the SCOPE1 trial in which females did not show a pronounced difference in OS from males following dCRT treatment.^29^ Other reports suggested a longer OS for females than males,^30^ but under different treatments.^31^, ^32^

Genomic profiling of ESCC tumors found 98% patients having amplification in one or more cancer‐relevant genes, as well as the co‐amplification of genes at adjacent chromosome locations, such as *CCND1/FGF19* and *TERC/SOX2/PIK3CA*. The amplification of *TERC* and *SOX2* was frequently observed in a variety of SCC, including oral and oropharyngeal,^33^ lung, esophagus,^34^ and cervical.^35^ However, despite their prevalence in cancers, those genes were not correlated with any of the survival parameters in this study.

Interestingly, *YAP1* amplification was significantly correlated with shorter LRFS, but not PFS and OS. In a subgroup of patients (male patients with stage II/III disease), of which the impact of sex and clinical stages was controlled, the difference in LRFS between *YAP1* amplification and wt groups was more pronounced, suggesting that *YAP1* is an independent prognostic marker for dCRT. Although the underlying mechanisms were not fully elucidated, our current understanding of the physical and pathological roles of YAP1 is supportive of this finding. YAP1 is the main downstream target of the Hippo pathway and acts as a transcriptional coactivator to regulate organ growth,^36^, ^37^ tissue homeostasis,^38^ and neoplasia,^28^, ^39^, ^40^, ^41^ primarily by interacting with TEAD transcription factors, which subsequently target a series of oncogenes and tumor suppressors to promote cell proliferation, transformation, migration, and invasion.^42^, ^43^ YAP1 overexpression has been identified in non‐small cell lung cancer, urothelial carcinoma of the bladder, and pancreatic cancer, and is consistently correlated with unfavorable clinical outcomes.^44^, ^45^, ^46^ *YAP1* amplification was also reported in 4%‐6% of ESCC patients,^23^, ^47^ and was suggested by independent in vitro studies to mediate chemo‐ and radioresistance by downregulating PTEN,^48^ and upregulating EGFR and CDK6 expression.^49^, ^50^ While local relapse is a major challenge for patients receiving dCRT, it is important to identify high‐risk patient groups for close monitoring. Our study identified that a subgroup of patients with *YAP1 CNV* amplification tended to have earlier local relapse, and therefore, may require more intensive clinical interventions. It is necessary to validate this finding in a larger clinical cohort, and improve the treatment benefits for such patients.

## AUTHOR CONTRIBUTIONS

Honghai Dai: Conceptualization, supervision, project administration, funding acquisition, methodology, investigation, and manuscript preparation, review, and editing. Yang W. Shao: Methodology, software, formal analysis, data curation, and manuscript preparation, review, and editing. Xiaoling Tong: Formal analysis, investigation, and manuscript preparation, review, and editing. Xue Wu: Investigation, and manuscript preparation, review, and editing. Jiaohui Pang: Investigation and manuscript preparation, review, and editing. Alei Feng: Resources, investigation, and manuscript preparation, review, and editing. Zhe Yang: Conceptualization, supervision, project administration, funding acquisition, methodology, investigation, and manuscript preparation, review, and editing.

## Data Availability

Genetic sequencing data are available upon request, certain restrictions may apply.
