# Supplementary material for: YAP1 amplification as a prognostic factor of definitive chemoradiotherapy in nonsurgical esophageal squamous cell carcinoma
Source: Cancer Med. 2019 Dec 18;9(5):1628–37. doi: 10.1002/cam4.2761 (PMC7050074; doi:10.1002/cam4.2761)
Supplement: Supplementary file 1 [file CAM4-9-1628-s001.docx]

***YAP1* amplification** **as a prognostic factor of definitive chemoradiotherapy in** **nonsurgical esophageal squamous cell carcinoma**

**Supplementary Information**


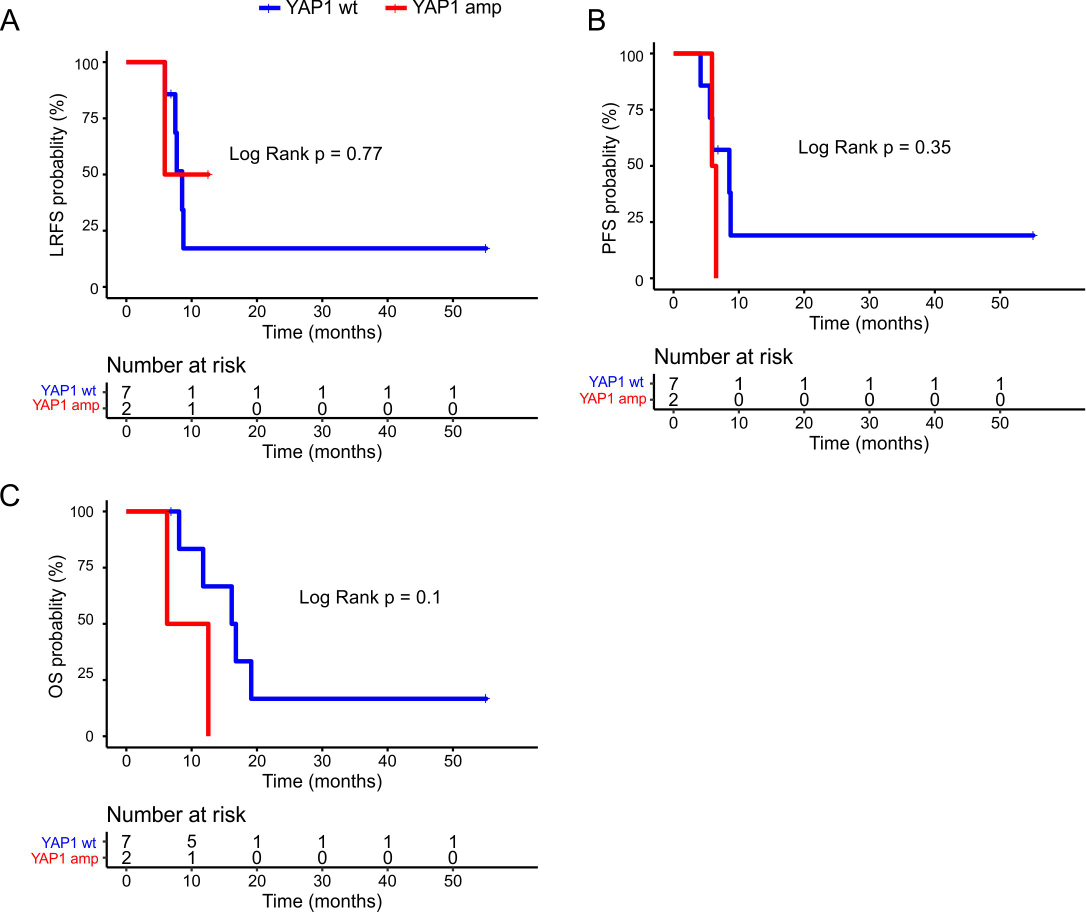


**Supplementary Figure 1.** Kaplan-Meier survival curves for local recurrence free survival (LRFS; panel A), progression free survival (PFS, panel B), and overall survival (OS, panel C) in female patients (n=9).
